# Mental Health and Social Care and Social Interventions

**DOI:** 10.3390/ijerph15112328

**Published:** 2018-10-23

**Authors:** Jed Boardman

**Affiliations:** 1 Institute of Psychiatry, Psychology and Neuroscience, King’s College, London SE5 8AF, UK; jedboard@atlas.co.uk; 2 Centre for Mental Health, Unit 2D21, Technopark, South Bank University, 90 London Road, London SE1 6LN, UK

In common parlance, the term ‘social’ is used in many senses ranging from the way society is organised to the rank or status someone has in society; to activities that involve meeting with other people; to the experience, behaviour and interaction of persons forming groups; and to promoting companionship and communal activities. At the microsocial level, it refers to the interaction of a person with others, their personal networks, their social world. At a macrosocial level, it relates to such constructs as social cohesion, shared values, economic and political environments and the attitudes of society that influence human behaviour. When applied to mental health conditions, the ‘social’ may be concerned with *social causation*, for example life events, disasters, urbanisation, unemployment, migration; *social consequences*, for example on the family, on public attitudes; *services* designed to provide assistance to people with mental health conditions (for example health and social services) or specific *psychosocial interventions*; and the *assessment and evaluation* of these services and interventions. This special edition is primarily concerned with the last two applications—services and interventions and their evaluation—but cannot help but stray into the social context and social consequences of mental health conditions.

Internationally, the trend for the provision and design of mental health services has been to move away from predominantly institutional settings to community settings. For example, over the past 50 years the United Kingdom has closed its large asylums and increased its community provision. In this special issue, Bouras et al.’s paper examines some of the consequences of this move and asks us to consider the concept of ‘Meta-community care’ for the development of future services in high-income countries. Their paper anticipates the focus of many of the other papers in this special issue, including stigma and discrimination, supported accommodation and employment, and socially focussed interventions for families and older adults. The stage of development of mental health services in high-income countries contrasts with that of many low- and middle-income countries discussed in Kohrt et al.’s comprehensive review of reviews that maps the community components of mental health programmes. Despite the differences in available resources, the components of services in these low- and middle-income nations mirror the types of social interventions, settings and social resources central to the delivery of mental health services internationally. These components include, among others, primary medical care, family, schools, mental health awareness, vocational rehabilitation, the use of lay counsellors and peer workers, as well as working closely with people who have lived experience of mental ill health. It appears that there is much to be gained from taking an international view of the delivery of mental health services. We have much to learn from one another.

A central concern for mental health services has been the types of outcomes that they aim to facilitate [1]. For many people with mental health conditions a full symptomatic recovery is not possible and even those with symptomatic improvement do not regain their full functioning or live a satisfactory life. The recovery movement has stressed not only the need for improvements in mental health services that are necessary to provide an environment that recognises the importance of the patients’ voice and their personal recovery, but also the need for political and societal change that recognises their rights to citizenship and social justice [2]. Ramon, in her paper on the place of social recovery, clarifies the differences between the overlapping categories of personal and social recovery and emphasises the importance of citizenship and the role of co-production in improving outcomes and status for people who experience mental ill health.

There has been increasing recognition of the role of people with lived experience in planning not only their own therapeutic activities, but also playing an integral part in developing mental and other health services [3]. This has been reflected in the development of person-centred approaches to professional practice [4] and in the use of the expertise that emerges from lived experience such as in the creation of peer workers [5]. Byrne et al. highlight the development of Peer Support Workers (PSWs) and report on the views of managerial staff in a variety of mental health organisations. Whilst uncovering generally positive views of the staff towards the employment of PSWs, Byrne et al. also recognise the mutual benefits of employing PSWs for service users and staff and the importance of providing good support and supervision for PSWs in their daily work.

The value of co-productive working with people with mental health conditions may also be extended to research settings. For example, many research funders specify that service users should be involved in the design and execution of research projects [6,7], and people with lived experience are increasingly involved in mental health research. In the UK there is a service user research workforce that is diverse, mature and highly skilled [6]. Keetharuth et al. provide an important example of co-production in the development of a Quality of Life measure (ReQol). This new and routinely used questionnaire provides a measure of personal recovery relevant to the experiences of people who use mental health services and has been developed through a co-productive process.

Taking a lead from the USA’s recovery centres [8], recovery leaders in the UK have facilitated the development of Recovery Colleges. There are now more than 77 Recovery Colleges in England providing courses for service users, carers and staff, all of which are underpinned by a co-production philosophy [9]. The paper by Stevens et al. reviews the development and working of these Colleges and examines the benefits of the arts-based courses provided in one Recovery College in southeast England. The use of creative media as a social intervention is emphasised in the paper and further explored by Schneider in relation to people experiencing dementia. Both papers suggest not only the value of the arts in providing aesthetic experiences but also their instrumental and therapeutic value. These approaches have the capacity to be used in the context of social prescribing [10].

Factors influencing a person’s personal and social recovery have been characterised as “something to do, somewhere to live and someone to love” [11]. Perhaps we could add to this list the need for an adequate amount of “money in the pocket” or necessary material resources. These financial, occupational and social network factors are essential drivers for the social inclusion of people with mental health conditions in society [12]. 

Ramon, in this issue, raises the problems of poverty in people with mental health conditions and the role of the provision of adequate financial support in reducing their isolation, maintaining their social networks and providing them with a sense of agency and mastery. Employment and housing are the subjects of the papers by Hutchinson et al. and McPherson et al. The report contained in McPherson et al.’s paper is part of a larger research programme designed to examine supported housing in the UK [13]. Research in the field of supported housing has been dogged by the lack of a clear taxonomy of supported housing, thus undermining attempts to clearly define the intervention variable. With the exception of the ‘Housing First’ approach [14], there is no convincing evidence for the efficacy of supported housing from randomised control trials (RCTs) and, given the heterogeneity of supported housing schemes, an RCT may not be feasible presently. The same, however, cannot be said of supported employment. For one approach, Individual Placement and Support (IPS), the evidence for its value is strong, with over 18 RCTs across the world providing positive results [15]. Despite this clear evidence, the same success has not been achieved in the implementation of IPS programmes [16]. Hutchinson et al. provide evidence for factors influencing the implementation of IPS in six sites in England. The sites were successful at getting people with severe and enduring mental health conditions into open employment, but less successful at sustaining their IPS services. One general factor known to promote the effectiveness of IPS is the degree to which the IPS model is put into practice (“Fidelity to the Model”), a model that promotes close collaboration between the vocational specialist, the service user and the mental health team: a person-centred approach.

Qualitative evidence for the value of supported housing also indicates the importance of a recovery-orientated approach to working and the quality of the environmental setting to be important in the success of housing support [17,18]. The importance of the immediate living environment is highlighted in the paper by Tyrer. This paper introduces Nidotherapy, an approach to systematically manipulating the immediate environment to better accommodate an individual’s metal health needs. This approach harks back to the older classic socially orientated studies of Brown and Wing and Goffman and the development of Therapeutic Communities and Milieu Therapy [19,20]. Again, the development of RCTs to assess Nidotherapy is challenging, but co-productive approaches seem to be central to its implementation.

In their paper discussing Family Group Conferencing (FGC) and its application to mental health, Schout and de Jong highlight the importance of the family environment. As with Open Dialogue [21], intervening in the family setting may have an empowering effect on individual and group solidarity. In their paper Schout and de Jong remind us that broader macro-environmental factors may have a significant effect at the individual and family level. They highlight the “constraints and strictures of the neoliberal welfare state” and the weakening of kinship ties as influencing family solidarity. 

The effects of the broader economic and political environment are also highlighted in the papers by Bouras et al. and Cummins, whilst Henderson and Gronholm focus on the corrosive effects of stigma and discrimination. We know that mental health problems themselves may impair individual functioning, but the disabilities experienced by people with mental health conditions, especially those with long-term conditions, are best understood with reference to a Social Model of Disability that takes into consideration contextual factors (environmental and personal factors) [22]. Stigma and Discrimination account for many of the environmental constraints placed on people with mental health conditions. Henderson and Gronholm conceptualise mental health-related stigma as a ‘wicked problem’, one that is complex and difficult to define, and leads to problems for the development of solutions at the interpersonal and structural level. They conclude that anti-stigma programmes, rather than being seen as impossible, remain important social interventions and require a broad range of approaches. Importantly, stigma and discrimination are everyone’s problem.

The final paper by Cummins reinforces the importance of the broader political and economic environment on the development and maintenance of mental health conditions and the development of effective services. The pursuit of policies of austerity after 2007 has been considered by some to be an experiment conducted by politicians, economists and ministers of finance, the effect of which has been to cut health and social services and social security spending—policies that have a profound effect on the vulnerable in society, including the rises in suicides seen in several countries [23]. 

The papers included in this special issue provide some examples of the social interventions that may benefit those with mental health problems, as well as highlighting some of the barriers to improving the development and efficacy of social interventions and services. The papers contained in the issue are a microcosm of many of the key issues relating to the development of mental health services, including expanding the scope of outcomes for people with mental health conditions beyond symptoms and functioning to embrace those of personal and social recovery; changing the relationships between those people who use services and those who provide them to incorporate a greater degree of co-productivity; engaging communities in mobilising community capital; focusing on improving the material and living conditions of people with mental health conditions; and reducing the toxic nature of the environment and economic policies.

## References

[1] Shepherd G., Boardman J., Rinaldi M., Roberts G. (2014). Supporting Recovery in Mental Health Services—Quality and Outcomes.

[2] Davidson L., Rakfeldt J., Strauss J. (2010). The Roots of the Recovery Movement in Psychiatry: Lessons Learned.

[3] Slade M. (2009). Personal Recovery and Mental Illness: A Guide for Mental Health Professionals.

[4] Ahmad N., Ellins J., Krelle H., Lawrie M. (2014). Person-Centred Care: From Ideas to Action. Bringing Together the Evidence on Shared Decision Making and Self-Management Support.

[5] Repper J., Carter T. (2011). A review of the literature on peer support in mental health services. J. Ment. Health.

[6] Patterson S., Trite J., Weaver T. (2014). Activity and views of service users involved in mental health research: UK survey. Br. J. Psychiatry.

[7] Ennis L., Wykes T. (2013). Impact of patient involvement in mental health research: Longitudinal study. Br. J. Psychiatry.

[8] Whitley R., Strickler D., Drake R.E. (2011). Recovery Centers for People with Severe Mental Illness: A Survey of Programs. Community Ment. Health J..

[9] Perkins R., Meddings S., Williams S., Repper J. (2018). Recovery Colleges 10 Years On.

[10] Bickerdike L., Booth A., Wilson P.M., Farley K., Wright K. (2017). Social prescribing: Less rhetoric and more reality. A systematic review of the evidence. BMJ Open.

[11] Dunn S. (1999). Creating Accepting Communities. Report of the Mind Inquiry into Social Exclusion and Mental Health Problems.

[12] Boardman J., Currie A., Killaspy H., Mezey G. (2010). Social Inclusion and Mental Health.

[13] Killaspy H., Priebe S., Bremner S., McCrone P., Dowling S., Harrison I., Krotofil J., McPherson P., Sandhu S., Arbuthnott M. (2016). Quality of life, autonomy, satisfaction, and costs associated with mental health supported accommodation services in England: A national survey. Lancet Psychiatry.

[14] Tsemberis S. (2010). Housing First: The Pathways Model to End Homelessness for People with Mental Illness and Addiction.

[15] Modini M., Tan L., Brinchmann B., Wang M.J., Killackey E., Glozier N., Mykletun A., Harvey S.B. (2016). Supported employment for people with severe mental illness: Systematic review and meta-analysis of the international evidence. Br. J. Psychiatry.

[16] Boardman J., Rinaldi M. (2016). Difficulties in implementing supported employment for people with severe mental health problems. Br. J. Psychiatry.

[17] Boardman J. (2016). More than Shelter. Supported Accommodation and Mental Health.

[18] Krotofil J., McPherson P., Killaspy H. (2018). Service user experiences of specialist mental health supported accommodation: A systematic review of qualitative studies and narrative synthesis. Health Soc. Care Community.

[19] Wing J.K., Brown G.W. (1970). Institutionalism and Schizophrenia. A Comparative Study of Three Mental Hospitals. 1960–1968.

[20] Goffman E. (1961). Asylums.

[21] Aaltonen J., Seikkula J., Lehtinen K. (2011). The Comprehensive Open-Dialogue Approach in Western Lapland: I. The incidence of non-affective psychosis and prodromal states. Psychos. Psychol. Soc. Integr. Approaches.

[22] World Health Organisation (2001). The International Classification of Functioning, Disability and Health (ICF).

[23] Stuckler D., Basu S. (2013). The Body Economic. Why Austerity Kills.

